# Harnessing host–virus evolution in antiviral therapy and immunotherapy

**DOI:** 10.1002/cti2.1067

**Published:** 2019-07-08

**Authors:** Steven M Heaton

**Affiliations:** ^1^ Department of Biochemistry & Molecular Biology Monash University Clayton VIC Australia

**Keywords:** antiviral, host‐oriented, host–virus interaction, information economy paradox, interferon, multifunctional host protein, neo‐virology, RNAi, vaccine

## Abstract

Pathogen resistance and development costs are major challenges in current approaches to antiviral therapy. The high error rate of RNA synthesis and reverse‐transcription confers genome plasticity, enabling the remarkable adaptability of RNA viruses to antiviral intervention. However, this property is coupled to fundamental constraints including limits on the size of information available to manipulate complex hosts into supporting viral replication. Accordingly, RNA viruses employ various means to extract maximum utility from their informationally limited genomes that, correspondingly, may be leveraged for effective host‐oriented therapies. Host‐oriented approaches are becoming increasingly feasible because of increased availability of bioactive compounds and recent advances in immunotherapy and precision medicine, particularly genome editing, targeted delivery methods and RNAi. In turn, one driving force behind these innovations is the increasingly detailed understanding of evolutionarily diverse host–virus interactions, which is the key concern of an emerging field, neo‐virology. This review examines biotechnological solutions to disease and other sustainability issues of our time that leverage the properties of RNA and DNA viruses as developed through co‐evolution with their hosts.

## Introduction

Most human‐infective viruses are RNA viruses, 94% of which harbour a single‐stranded RNA (ssRNA) genome.^1^ These include established pathogens such as HIV and dengue virus (DenV), most high‐profile emerging pathogens this decade [e.g. Zika virus (ZikV), SARS‐coronavirus (SARS‐CoV) and avian influenza], re‐emerging pathogens including measles virus (MV) and every pathogen prioritised in the recent WHO R&D Blueprint.^2^ Furthermore, climate change‐related factors are likely to drive changes in future dispersion or transmission of viruses including mosquito‐borne viruses such as DenV and ZikV.^3^ The disease burden associated with many of the 214 human‐infective RNA virus species is large and growing, yet only five have US Food and Drug Administration (FDA)‐approved antivirals available and nearly all target virus proteins (Table 1).

**Table 1:** Types and targets of current Food and Drug Administration‐approved antiviral drugs

Name	Type	Approved	Target	Virus/es
Cytarabine	Small molecule	1969	Host	HSV
Interferon alfa‐2b	Protein	1997	Host	HBV, HCV
Interferon alfacon‐1	Protein	1997	Host	HCV
Peginterferon alfa‐2b	Protein	2001	Host	HCV
Peginterferon alfa‐2a	Protein	2002	Host	HBV, HCV
Ribavirin	Small molecule	2002	Host	HCV
Enfuvirtide	Peptide	2003	Host	HIV
Maraviroc	Small molecule	2007	Host	HIV
Idoxuridine	Small molecule	1963	Virus	HSV
Amantadine	Small molecule	1966	Virus	IAV
Vidarabine	Small molecule	1976	Virus	HSV, VZV
Zidovudine	Small molecule	1987	Virus	HIV
Ganciclovir	Small molecule	1989	Virus	CMV
Foscarnet	Small molecule	1991	Virus	HSV
Zalcitabine	Small molecule	1992	Virus	HIV
Stavudine	Small molecule	1994	Virus	HIV
Rimantadine	Small molecule	1994	Virus	IAV
Saquinavir	Small molecule	1995	Virus	HIV
Lamivudine	Small molecule	1995	Virus	HIV, HBV
Trifluridine	Small molecule	1995	Virus	HSV
Valaciclovir	Small molecule	1995	Virus	HSV, VZV
Cidofovir	Small molecule	1996	Virus	CMV
Didanosine	Small molecule	1996	Virus	HIV
Indinavir	Small molecule	1996	Virus	HIV
Nevirapine	Small molecule	1996	Virus	HIV
Ritonavir	Small molecule	1996	Virus	HIV
Penciclovir	Small molecule	1996	Virus	HSV
RespiGam	Plasma antibody	1996	Virus	RSV
Delavirdine	Small molecule	1997	Virus	HIV
Nelfinavir	Small molecule	1997	Virus	HIV
Famciclovir	Small molecule	1997	Virus	HSV
Acyclovir	Small molecule	1997	Virus	HSV, VZV
Fomivirsen	Oligonucleotide	1998	Virus	CMV
Abacavir	Small molecule	1998	Virus	HIV
Efavirenz	Small molecule	1998	Virus	HIV
Viroptic	Small molecule	1998	Virus	HSV
Palivizumab	Humanised mAb	1998	Virus	RSV
Amprenavir	Small molecule	1999	Virus	HIV
Oseltamivir	Small molecule	1999	Virus	IAV, IBV
Zanamivir	Small molecule	1999	Virus	IAV, IBV
Lopinavir	Small molecule	2000	Virus	HIV
Docosanol	Small molecule	2000	Virus	HSV
Valganciclovir	Small molecule	2001	Virus	CMV
Tenofovir	Small molecule	2001	Virus	HIV, HBV
Adefovir	Small molecule	2002	Virus	HBV
Atazanavir	Small molecule	2003	Virus	HIV
Emtricitabine	Small molecule	2003	Virus	HIV
Fosamprenavir	Small molecule	2003	Virus	HIV
Entecavir	Small molecule	2005	Virus	HBV
Tipranavir	Small molecule	2005	Virus	HIV
Telbivudine	Small molecule	2006	Virus	HBV
Darunavir	Small molecule	2006	Virus	HIV
Raltegravir	Small molecule	2007	Virus	HIV
Etravirine	Small molecule	2008	Virus	HIV
Boceprevir	Small molecule	2011	Virus	HCV
Telaprevir	Small molecule	2011	Virus	HCV
Rilpivirine	Small molecule	2011	Virus	HIV
Simeprevir	Small molecule	2013	Virus	HCV
Sofosbuvir	Small molecule	2013	Virus	HCV
Dolutegravir	Small molecule	2013	Virus	HIV
Peramivir	Small molecule	2014	Virus	IAV
Daclatasvir	Small molecule	2015	Virus	HCV
Letermovir	Small molecule	2017	Virus	CMV
Doravirine	Small molecule	2018	Virus	HIV
Ibalizumab	Humanised mAb	2018	Virus	HIV
Baloxavir	Small molecule	2018	Virus	IAV, IBV
Tecovirimat	Small molecule	2018	Virus	Smallpox

Approved combination therapies excluded.

CMV, cytomegalovirus; HBV, hepatitis B virus; HCV, hepatitis C virus; HIV, human immunodeficiency virus; HSV, herpes simplex virus; IAV, influenza A virus; IBV, influenza B virus; mAb, monoclonal antibody; RSV, respiratory syncytial virus; VZV, varicella‐zoster virus.

While virus‐oriented approaches are efficacious, the genetic diversity of viruses often restricts such treatments to particular species or serotypes (Table 1). Furthermore, these antivirals are often costly and are ultimately susceptible to escape mutant selection. Simple point substitutions are often responsible for treatment failure,^4^, ^5^ while fitness costs associated with harbouring these substitutions may be trivially absorbed by the escaped strain upon accumulating compensatory adaptations.^6^ Tenofovir is an example of a highly effective single‐regimen treatment for chronic hepatitis B infection, a retro‐transcribing virus characterised by considerable genetic heterogeneity, by simultaneously imposing potent viral suppression, a high barrier for escape and reduced replicative fitness of escape strains. Despite these synergising effects, complex escape mutants harbouring multiple point substitutions in the viral reverse transcriptase have recently emerged.^7^ One way of enhancing treatment efficacy while minimising viral escape is to deploy existing antivirals as combination therapies, a strategy used extensively in current HIV (e.g. tenofovir/emtricitabine) and hepatitis C virus (HCV) treatment regimens.^4^, ^5^ While increasing the number of combinations increases the height of the escape barrier, proportional increases in treatment costs, adverse effects and counterindications make this strategy one of ever compounding challenges that ultimately remains exposed to the core problem of viral resistance. Treatment failure and the continuous need for the development of additional therapies are the realised costs of playing into such ‘strengths’ of virus evolution.

As obligate intracellular parasites, all viruses must subvert key resources of permissive hosts in order to replicate.^8^ Subverting multifunctional host proteins can confer significant fitness advantages by enabling RNA viruses to efficiently execute multiple steps in their replication strategy. Over time, these features are likely to be conserved within lineages and serve as foci of evolutionary convergence for viruses with a similar host range, while purifying selection eliminates steps rendered less efficient. Nevertheless, ideal targets of pathogenic viruses include those that are also vital to the host, thereby limiting its options for antiviral adaptation and driving more costly evolutionary innovation on its part. Similarly, the potential for adverse effects limits options for targeting such host proteins therapeutically.

Therapeutic drug availability, together with recent advances in areas including immunotherapy and precision medicine, is beginning to alleviate such constraints on host‐oriented approaches. Significantly, many of these technologies arose through examining evolutionarily diverse host–virus and immune interactions, which are being increasingly uncovered with the advent of mass next‐generation genome sequencing and machine learning‐assisted metagenomic analysis technologies. Furthermore, such interactions are increasingly found to perform crucial roles throughout our biosphere.^9^, ^10^, ^11^ As was once the case for the CRISPR/Cas bacterial immune system proteins now used in genome editing,^12^ these host–virus interactions often employ unique proteins of unknown function.^10^, ^13^, ^14^ This review examines how host–virus evolution may be leveraged towards solving disease and sustainability issues of our time. Multitasking or multifunctional host proteins as antiviral therapeutic targets, methods for targeting such proteins, vaccine design and neo‐virology as an emerging source of biotechnological innovation, will be discussed.

## Exploiting the information economy paradox in RNA virus evolution

**Figure 1:**
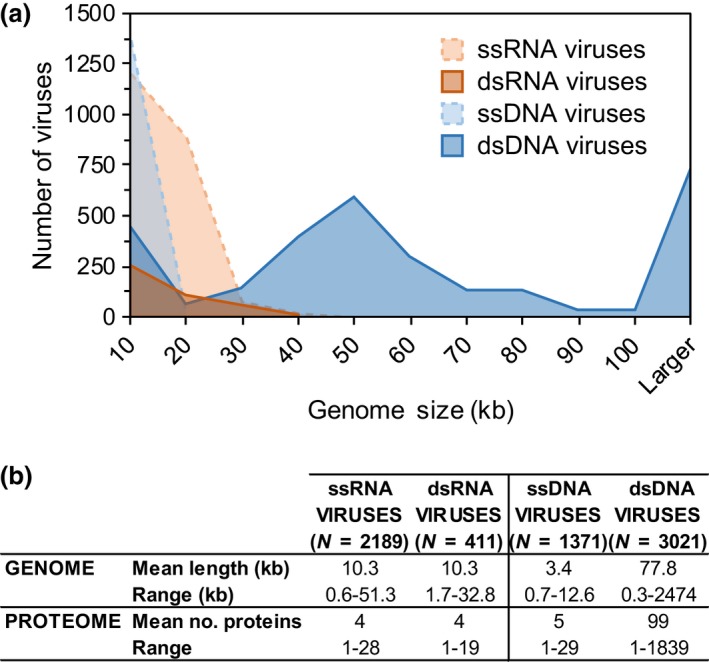
Genome and proteome size distribution of RNA versus DNA viruses. Data compiled using the NCBI Viral Genomes Resource,^116^ taxonomic ID 10239, accessed March 2019. Incomplete, unclassified and sub‐viral genomes excluded. **(a)** Histogram of virus genome sizes. Orange = RNA viruses; blue = DNA viruses; dashed line = single‐stranded genomes; solid line = double‐stranded genomes. **(b)** Range and average genome and proteome sizes of viruses plotted in **a**.

RNA and retro‐transcribing virus genomes are highly versatile, with errors occurring 2–4 orders of magnitude more frequently than in high‐order eukaryotes.^15^ This, combined with their rapid replication cycle, imbues such viruses with two key strengths: enormous genetic diversity and rapid escape mutant selection. Nevertheless, this same process that enables remarkable genome plasticity also appears to limit the incorporation of new information with which to achieve more favorable host manipulation. There exists an inverse relationship between viral genome size and mutation rate, with large coronaviruses the only known RNA virus family to possess 3′‐exonuclease proofreading activity.^16^ Thus, the probability of acquiring a lethal mutation increases as a function of both polymerase infidelity and genome size. This suggests lengthening the genome to accommodate a larger repertoire of gene products with which to better manipulate the host comes with considerable fitness trade‐offs for RNA viruses. Indeed, excepting extremely small circular ssDNA viruses, RNA virus genomes are typically far shorter than DNA virus genomes in both average size (10.3 vs. 77.8 kb, respectively) and maximal size (51.3 vs. 2474 kb) and encode fewer proteins (1–28 vs. 1–1839; Figure 1). As a result, while various RNA viruses tolerate certain gene substitutions (e.g. recombinant reporter strains and segmented genome viruses),^17^ most are poorly tolerant of additions of new genetic information. Correspondingly, mutagenic nucleoside analogues exhibit broad‐spectrum antiviral activity by increasing the error rate in genome synthesis for nascent viral particles.^18^ This effectively reduces the optimal genome length of the virus for productive infection to below the threshold of viability, thereby driving the population to extinction. Therefore, while the core ‘strengths’ in RNA virus evolution arise because of the nature of their genetic material and its error‐prone mode of replication, these appear intractably coupled to limits on the size of information available to subvert their more complex hosts.

RNA virus evolution attempts to resolve this information economy paradox by extensively employing functional genomic secondary structures and noncoding regions, genome segmentation, compression (e.g. RNA editing, overprinting and frameshift reading) and gene product pleiotropy or multifunctionality (e.g. intrinsically disordered proteins).^17^, ^19^, ^20^, ^21^ Yet another way is to manipulate host cell factors that are themselves multi‐interacting or multifunctional ‘hubs’ of cellular activity,^22^ whereby a single viral gene product subverts a single host factor to achieve net favorable control over numerous cellular processes. This can enable the virus to extract maximum utility from its informationally limited genome at minimal informational cost. Multiplying this effect across several viral and/or host gene products may enable the virus to extract a substantial ‘return on investment’ in terms of replicative fitness.

Despite facilitating viral infectivity, these solutions to the information economy paradox cut both ways. Imbricated dependency on multifunctional host proteins for various key replication steps creates vulnerabilities that may be exploited for highly efficacious antiviral therapies. For example, denying such host proteins to the virus may disrupt multiple key elements in its replication strategy. Where these proteins represent foci of evolutionary convergence, such therapies may yield robust, broad‐spectrum antiviral activity. The degree of innovation required to circumvent such a therapy and, especially in the case of RNA viruses, the informational barrier to realising this innovation may be high. Escape mutants that emerge may be forced to overcome multiple deficiencies simultaneously and suffer compounding fitness penalties in the process. Furthermore, host‐oriented approaches present a much larger list of potential therapeutic targets than the dozen or so gene products produced by most human‐infective RNA viruses, many of which are challenging targets in the first instance because of genetic diversity and intrinsic protein disorder.^19^ Given the higher fidelity of DNA versus RNA replication, host therapeutic targets may prove resilient to certain mechanisms of viral resistance. Altogether, these advantages may reduce antiviral development costs over the long term, allowing for greater treatment accessibility and faster development of future therapies. But which host proteins are sufficiently multifunctional? And how might these present viable therapeutic substrates?

## Multifunctional host proteins as potential antiviral targets

**Figure 2:**
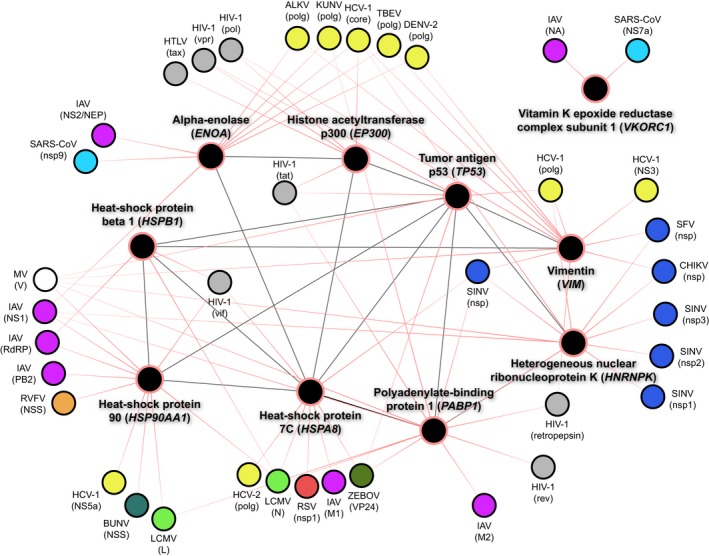
Interaction map of the 10 most multifunctional human proteins targeted most frequently by viruses. The 282 most multifunctional human proteins^117^ were used to interrogate available protein interaction data with the VirHostNet tool.^118^ Top 10 virus‐interacting multifunctional host proteins represented in black filled circles and labelled in bold type. Black lines depict host–host protein interactions, and red lines depict host–virus protein interactions. ssRNA viral protein interacting partners represented as coloured filled circles (clockwise from the top: yellow = Flaviviridae, purple = Orthomyxoviridae; light blue = Coronaviridae; dark blue = Togaviridae; grey = Retroviridae; dark green = Filoviridae; red = Pneumoviridae; light green = Arenaviridae; teal = Peribunyaviridae; orange = Phenuiviridae; white = Paramyxoviridae). The complete data set is shown in Supplementary table 1.

To canvass potential therapeutic targets, the 282 most multifunctional human proteins identified to date were used to interrogate available protein interaction data (Figure 2). Strikingly, 77% (216/282) of these have been experimentally determined to interact with at least one viral protein (Supplementary table 1). Of these, 74% interact with at least one ssRNA viral protein, highlighting how multifunctional host proteins represent key ssRNA viral manipulation targets. The three highly multifunctional host proteins targeted by the greatest diversity of ssRNA virus families are as follows: heat‐shock protein 90a (HSP90a), HSP7C and polyadenylate‐binding protein 1 (PABP1; Figure 2). These proteins appear to serve as key drivers of convergence between diverse human‐infective viruses, suggesting these are potential targets of broad‐spectrum antivirals. Additional targets of significant interest include alpha‐enolase, heat‐shock protein beta‐1 (HSPB1, also termed HSP27), heterogeneous nuclear ribonucleoprotein K (hnRNPK), histone acetyltransferase (HAT) p300, vimentin, vitamin K epoxide reductase complex subunit 1 (VKORC1) and tumor antigen p53 (Figure 2 and Table 2). Diverse RNA viruses targeting these proteins, as well as possible therapeutic avenues, are discussed below.

**Table 2:** Approved, investigational or experimental bioactive compounds for the 25 most multifunctional host proteins targeted most frequently by viruses

Rank	UniProt	Multitasking host proteins	Approved, investigational or experimental host protein‐targeting drugs	Host protein‐targeting RNA viruses
1	P06733	Alpha‐enolase	Artenimol, AP‐III‐a4/ENOblock	AlkV, DenV, HCV, IAV, KunV, SARS‐CoV, TBEV
2	P04637	Tumor antigen p53	Acetylsalicylic Acid, AZD 3355, 1‐(9‐ethyl‐9H‐carbazol‐3‐yl)‐*N*‐methylmethanamine	HCV, HIV‐1, MV, RV, ZEboV
3	P61978	Heterogeneous nuclear ribonucleoprotein K	Artenimol, Bortezomib, Phenethyl Isothiocyanate	ChikV, HCV, HIV‐1, IAV, MV, SFV, SinV, ZEboV
4	Q9BQB6	Vitamin K epoxide reductase complex subunit 1	Menadione, Warfarin	IAV, SARS‐CoV
5	Q09472	Histone acetyltransferase p300	Anacardic Acid, Curcumin, Demethoxycurcumin, Garcinol, Histone Acetyltransferase Inhibitor II, A‐485, C646, L002, Lys‐CoA	HCV, HIV‐1, HTLV
6	P04792	Heat‐shock protein beta‐1	Apatorsen, Artenimol, J2, Phenethyl Isothiocyanate	HIV‐1, IAV, LCMV
7	P07900	Heat‐shock protein 90‐alpha	Aminoxyrone, Ganetespib, Geldanamycin, Luminespib, Onalespib, Retaspimycin, Tanespimycin/17‐AAG, AUY922, BIIB021, IPI‐493, SNX‐5422, STA‐9090, XL888	BunV, HCV, HIV‐1, IAV, LCMV, MV, RVFV
8	P08670	Vimentin	Artenimol, Calyculin A, Epigallocatechin Gallate, Okadaic Acid, Phenethyl Isothiocyanate, Salinomycin, SB431542, Withaferin A	AlkV, ChikV, DenV, HCV, HIV‐1, MV, SFV, SinV, TBEV
9	P11940	Polyadenylate‐binding protein 1	Artenimol	HIV‐1, IAV, KunV, LCMV, MV, ReoV, SinV, ZEboV
10	P11142	Heat‐shock cognate 71 kDa protein	Artenimol, Dasatinib	HCV, HIV‐1, IAV, LCMV, RSV, SinV, ZEboV
11	Q93062	RNA‐binding protein with multiple splicing		HTLV, IAV
12	P12004	Proliferating cell nuclear antigen	Acetylsalicyclic Acid, Liothyronine	HIV‐1, IAV, ZEboV
13	P08238	Heat‐shock protein 90‐beta	Geldanamycin, Tanespimycin/17‐AAG, CNF1010, SNX‐5422	DenV, HCV, KunV, RVFV
14	Q14160	Protein scribble homolog		AlkV, HTLV, IAV, RabV, TBEV
15	P04406	Glyceraldehyde‐3‐phosphate dehydrogenase	Adenosine‐5‐Diphosphoribose, Artenimol, Thionicotinamide‐Adenine‐Dinucleotide, Xanthinol, 4‐(2‐Aminoethyl)Benzenesulfonyl Fluoride	HeV, HIV, LCMV
16	Q8N448	Ligand of numb protein X2		HTLV, IAV
17	Q08379	Golgin subfamily A member 2		HCV, RSV
18	P62258	14‐3‐3 protein epsilon	Fusicoccin, Phenethyl Isothiocyanate	HCV, HIV‐1, IAV, NiV, SinV
19	P22736	Nuclear receptor subfamily 4 group A member 1		HCV
20	P27986	PI3K regulatory subunit alpha	Enzastaurin, Isoprenaline, Wortmannin, SF1126	HEV, HIV, IAV
21	P63279	Small ubiquitin‐like modifier‐conjugating enzyme Ubc9		DenV, HIV‐1
22	Q99816	Tumor susceptibility gene 101 protein		HCV, HEV, HeV, HIV‐1, HIV‐2, HSRV, HTLV
23	Q13200	26S proteasome non‐ATPase regulatory subunit 2		DenV, HCV, HIV‐1, IAV, MV
24	P14618	Pyruvate kinase PKM	Artenimol	DenV, HCV, IAV, LCMV
25	Q9UNE7	E3 ubiquitin–protein ligase CHIP		DenV, HIV‐1, IAV

Proteins ranked according to total number of viral interacting partners.

RNA viruses shown: AlkV, Alkhumra haemorrhagic fever virus; BunV, Bunyamwera virus; ChikV, chikungunya virus; DenV, dengue virus; HCV, hepatitis C virus; HEV, hepatitis E virus; HeV, Hendra virus; HIV, human immunodeficiency virus; HSRV, human spumaretrovirus; HTLV, human T‐lymphotropic virus; IAV, influenza A virus; KunV, Kunjin virus; LCMV, lymphocytic choriomeningitis virus; MV, measles virus; NiV; Nipah virus; RabV, rabies virus; ReoV, reovirus; RSV, respiratory syncytial virus; RVFV, Rift Valley fever virus; SARS‐CoV, severe acute respiratory syndrome‐coronavirus; SFV, Semliki Forest virus; SinV, Sindbis virus; TBEV, tick‐borne encephalitis virus; ZEboV, Zaire Ebola virus.

### Measles virus (MV)

Measles virus is an extremely contagious and virulent pathogen undergoing a recent global resurgence. The non‐structural V protein targets the single largest number of highly multitasking human proteins: HSP90a, PABP1, vimentin, hnRNPK and p53 (Figure 2). In addition to V's established roles in suppressing multiple components of host interferon (IFN) signalling,^23^ these interactions may allow MV to interface with the cytoskeleton (vimentin) and subvert numerous host cell processes including cell cycle (p53, hnRNPK), protein translation (PABP1), RNA metabolism (PABP1, hnRNPK), and transcription and protein expression (HSP90a). V is produced by editing of the *P* gene transcript, which also overlaps with the *C* gene. The largest number of amino acid substitutions between wild‐type MV and attenuated vaccine strains occurs within the *P*/*V*/*C* gene region,^24^ suggesting changes in this region serve an important basis for natural attenuation. Since attenuated MV strains possess very limited capacity for reversion,^24^ MV strains engineered to harbour defects in V binding to these host proteins may be suitable designer vaccine candidates. As discussed in the following sections, re‐purposing existing host‐targeting bioactive compounds as antivirals may also yield therapeutic results.

### Human immunodeficiency virus (HIV)

The HIV‐1 accessory protein viral infectivity factor (Vif), also crucial in suppressing host immunity,^25^ targets all three highly multitasking heat‐shock proteins HSP90a, HSPB1/HSP27 and HSP7C (Figure 2). This suggests subversion of the cellular protein quality control pathways or HSP‐mediated gene expression is vital in the HIV‐1 replication cycle. Accordingly, HSP90 inhibitors 17‐AAG/tanespimycin and AUY922 (Table 2) were recently shown to inhibit HIV‐1 transcription and suppress viral rebound in a humanised mouse model.^26^ Although these and other HSP90 inhibitors have encountered efficacy and toxicity issues during clinical trials as anticancer therapies, aminoxyrone is novel, first‐in‐class HSP90 inhibitor that appears to alleviate both issues.^27^ Its efficacy as an antiviral or antiretroviral therapeutic has yet to be studied. Inhibitors of HSPB1 and HSP7C (Table 2) represent additional avenues for effective host‐oriented antiretroviral therapies that have also yet to be explored.

HIV‐1 tat (transactivating regulatory protein), which is required for efficient viral gene transcription,^28^ targets PABP1, p53 and HAT p300 (Figure 2). The latter protein is further manipulated by two additional HIV‐1 proteins, viral protein R (Vpr) and Pol (DNA polymerase), as well as the transactivating regulatory protein (Tax) of human T‐lymphotropic virus (HTLV; Figure 2), plausibly representing a conserved mechanism of host subversion. HATs and histone deacetylases (HDACs) are crucial effectors in epigenetic modulation of gene expression, connecting a large number of cell signalling inputs with transcriptional outputs through histone post‐translational modifications.^29^ HAT and HDAC inhibitors have been shown to suppress viral transcription (‘kill’) and re‐activate latent virus (‘shock‐and‐kill’), respectively,^30^, ^31^ suggesting epigenetic remodulation of host gene activity is exceptionally important in the replication strategy of HIV‐1 and other retroviruses. Alternatively, retroviral proteins may block or usurp the enzymatic activity of HAT p300 to direct acetylation of viral or other host proteins, which has been suggested for Tat.^32^ The interactions between HAT p300 and HIV‐1 proteins Tat, Vpr and Pol (Figure 2), together with the large number of HAT p300 inhibitors currently available (Table 2), expand the possibilities for targeting HAT p300 in host‐oriented antiretroviral therapies.

### Influenza A virus (IAV)

Influenza A virus (IAV) engages four gene products to manipulate two highly multifunctional host proteins. HSP90a is targeted by NS1 (virulence factor), PB2 (transcription and capping) and RNA‐dependent RNA polymerase (RdRP), while alpha‐enolase is targeted by NS1 as well as NS2/NEP (vRNP nuclear export; Figure 2). The NS1 proteins of avian influenza strain H5N1 as well as H3N2 reportedly bind HSP90 to modulate caspase‐mediated apoptosis.^33^ In addition to impairing HIV‐1 replication,^30^, ^31^ HSP90 inhibitors reportedly inhibit IAV replication without apparent cytotoxicity *in vitro*.^34^ It will be of interest to examine whether these effects translate *in vivo* using next‐generation HSP90 inhibitors^27^ (Table 2).

Influenza A virus, together with SARS‐CoV and multiple flaviviruses, also targets alpha‐enolase, an enzyme with roles in glycolysis, cell growth and immunity (Figure 2). A novel inhibitor, AP‐III‐a4 (ENOblock), was recently developed with the interesting property of blocking the non‐glycolytic functions of alpha‐enolase (Table 2). While this drug shows promise in treating obesity in animal models,^35^ its antiviral effects remain unexplored. This warrants further study as a potential host‐oriented antiviral approach to IAV and other viral infections.

### Togaviruses

Similarly, by committing four of its proteins to manipulating hnRNPK (Figure 2), Sindbis virus (SinV) reveals this multifunctional host protein to be crucial in its replication strategy. Co‐immunoprecipitation experiments suggest hnRNPK associates with the SinV polyprotein processing products nsp1 (methyl/guanylyltransferase), nsp2 (helicase/protease) and nsp3 (regulatory component) and may be required for viral transcription.^36^ Other *Togaviridae* members including Semliki Forest virus (SFV) and chikungunya virus (ChikV) also manipulate hnRNPK (Figure 2), suggesting this host protein serves multiple, evolutionarily conserved roles in togavirus replication. While this hints at a potential therapeutic route against togaviruses, there are currently no selective hnRNPK‐targeting drugs available (Table 2). hnRNPK is also tumor suppressor, with mutated or constitutively downregulated hnRNPK being associated with tumorigenesis.^37^ Nevertheless, short‐term therapeutic targeting of hnRNPK as an antiviral strategy has yet to be explored.

Viral infection often induces cytoskeletal remodelling, resulting in cytopathic morphologies including syncytia and tumor‐like aggregates. Cells treated with actin depolymerising agents such as cytochalasin D show drastic reductions in production of numerous viruses,^38^ although such agents, by their nature, have limited therapeutic applicability. Vimentin, a component of intermediate filaments, is manipulated by SinV, SFV and ChikV, together with MV as previously mentioned, and the flaviviruses DenV, HCV, tick‐borne encephalitis virus (TBEV), Alkhumra haemorrhagic fever virus (AlkV) and Kunjin virus (KunV)/West Nile virus (WNV; Figure 2). Vimentin is reported to play a key role in replication complex assembly and modulating viral protein expression levels in DenV and HCV infection, respectively.^39^, ^40^ Withaferin D targets the soluble form of vimentin^41^ (Table 2) and has anticancer properties, although the effects of vimentin‐targeting drugs in the context of infection have yet to be extensively studied.

### Flaviviruses

Numerous flaviviruses including DenV, HCV, TBEV, AlkV and KunV/WNV manipulate alpha‐enolase (Figure 2), an enzyme with many functions including catalysing the penultimate step in ATP synthesis via glycolysis. While viruses often re‐program cellular metabolic pathways, DenV drastically increases the rate of glycolysis to support its own replication. Metabolic acidosis is often associated with severe disease and may correlate with the subcellular redistribution of viral proteins to further compromise host stress responses.^42^ Thus, DenV‐infected patients who are simultaneously hyperglycaemic (e.g. diabetics) are at greater risk of severe disease.^43^ Accordingly, inhibiting glycolysis impairs replication of DenV and other flaviviruses *in vitro*.^44^, ^45^ In this context, it would be of interest to examine whether alpha‐enolase inhibitors such as AP‐III‐a4/ENOblock (Table 2), which blocks the non‐glycolytic functions of alpha‐enolase as mentioned previously, may block subversion of this key enzyme by flaviviruses *in vivo*.

### Other targets

One unexpected multiple viral target is VKORC1, an enzyme highly expressed in liver and crucial in activating blood clotting factors.^46^ While relatively fewer RNA viruses target VKORC1 (Figure 2), it is targeted by several DNA viruses including the hepatotropic Epstein–Barr virus. It would be of interest to examine what effects, if any, infection by such viruses have in the context of treatment with vitamin K or VKORC1‐targeting drugs such as warfarin (Table 2).

While not themselves multifunctional, ubiquitin and ubiquitin‐like modifiers undergo covalent and non‐covalent association with other proteins and exert plethoric effects on their function, abundance or subcellular distribution. Thus, manipulating the ubiquitin and ubiquitin‐like post‐translational modification machinery, or the proteasome itself, also enables viruses to subvert multiple cellular processes.^8^ Indeed, most multitasking host proteins examined here undergo extensive post‐translational modification.^47^ Numerous multitasking proteins of the ubiquitin–proteasome system, as well as ubiquitin and ubiquitin‐like modifiers, are key targets of multiple viruses. These targets include the proteasome regulatory subunit PSMD2; the E3 ubiquitin–protein ligase CHIP, which modulates the activity of numerous protein chaperones including HSP90^48^; and the small ubiquitin‐like modifier‐conjugating enzyme Ubc9, which regulates numerous cellular functions including cell cycle by modifying p53^49^ (Table 2 and Supplementary table 1). Numerous inhibitors of the proteasome, ubiquitin E1, E2 or E3 enzymes and deubiquitinating enzymes are currently in clinical trials or approved for use as anticancer agents.^50^ Therapeutically modulating the ubiquitin–proteasome system may present an indirect method of targeting multitasking proteins that are otherwise ‘undruggable’ at present (Table 2).

## Challenges and strategies in targeting multifunctional host proteins

Therapeutically targeting host proteins that converge various cellular processes can elicit unwanted effects. This challenge is familiar to the very viruses that exploit such proteins in the first instance, yet their success also implies its soundness as an antiviral strategy. Illustrating that multifunctional host proteins can be ‘druggable’, 48% of the 694 human multitasking proteins annotated by Franco‐Serrano *et al*.^51^ are already targets of known compounds, compared with 9.8% of all 26 199 human proteins listed in UniProt. This also suggests potential for re‐purposing existing drugs for host‐oriented antiviral therapy (Table 2).

Since viruses typically infect and replicate best in only a limited set of host tissues, delivering therapies to specific tissues may mitigate adverse effects. Synthetic lipid nanoparticles (LNPs) protect and deliver small RNAs for RNAi‐based therapy as well as synthetic vaccines and bioactive compounds,^52^, ^53^, ^54^, ^55^, ^56^ while modified viruses or virus‐like particles deliver RNA interference (RNAi)‐based therapies as well as nucleases and DNA for gene therapy.^57^, ^58^ Despite additional challenges as outlined below, these technologies are currently applied to deliver specific treatments for viral infection, cardiovascular disease, inherited genetic disorders and cancer immunotherapy in animal models and humans.^52^, ^53^, ^54^, ^55^, ^56^, ^57^

### RNA‐based therapies

#### miRNA and siRNA biogenesis

The last eukaryote common ancestor likely possessed an RNAi system utilising endogenous or exogenous noncoding RNAs (ncRNAs) and an RdRP.^59^ However, host–pathogen interactions have shaped RNAi utilisation throughout eukaryote diversification. Budding yeasts, including the prototypical *Saccharomyces* *cerevisiae*, harbouring endemic dsRNA viruses of the Totiviridae family lost RNAi while other yeasts lacking such viruses retained RNAi.^60^ With the rise of jawed vertebrates, RdRP was lost while IFN was gained, enabling large, complex life to coordinate multifurcated, system‐wide responses to infection and eventually supplanted RNAi as the primary antiviral defence mechanism in animals.^61^, ^62^ Nevertheless, ncRNAs continue to perform crucial roles in fundamental mammalian cellular processes including pre‐mRNA processing via spliceosomes (e.g. small nuclear RNA; snRNA). Small interfering RNA (siRNA) and microRNA (miRNA) are those most commonly applied in current RNAi biotechnology.

In addition to their sequence and tissue specificity, ncRNA function is determined by their expression level, post‐transcriptional processing and modification, protein interacting partners and subcellular compartmentalisation, as outlined below. miRNA is selectively expressed in all human tissues, with 1917 miRNAs currently annotated in the miRBase database predicted to control transcription of > 60% of human protein‐coding genes.^63^, ^64^ In the nucleus, primary miRNA transcripts are processed into 60–80 nt pre‐miRNA by the Microprocessor complex comprising the RNase‐III enzyme Drosha and DGCR8, the latter of which also associates with exosomes^65^ which play crucial roles in RNA processing and surveillance. Alternatively, mirtron miRNAs are spliced directly from introns by the spliceosome, independently of Drosha.^66^ Exportin‐5 transports pre‐miRNA into the cytosol, while a Drosha‐independent miRNA subset is reportedly transported in an exportin‐1‐/CRM1‐dependent manner.^67^

In the cytosol, the RNase‐III enzyme Dicer excises ~ 20–23 nt double‐stranded miRNA as well as endogenous and exogenous siRNA fragments.^68^ All RNase‐III cleavage products are characterised by a 2 nt 3′ overhang together with 5′‐monophosphorylated and 3′‐hydroxyated ends. The strand with the thermodynamically more stable 5′ end is usually degraded, while the remaining ssRNA guide strand is loaded onto Argonaute proteins. Together, these ribonucleoproteins form the RNA‐induced silencing complex (RISC).^69^ The endoribonuclease activity of Ago2 cleaves the target RNA to achieve post‐transcriptional repression,^70^ while other Argonaute proteins repress expression through non‐degradative mechanisms.

Exosomes and the exoribonuclease XRN1 are both required for full degradation of RISC cleavage products in *Drosophila*.^71^ Intriguingly, XRN1, which serves as a crucial viral restriction factor in totivirus‐harbouring (i.e. RNAi‐deficient) yeasts,^72^ and exosome core components RRP41, which associates with DGCR8,^65^ and RRP42 are identified here as among the most highly multitasking proteins most frequently manipulated by human‐infective viruses. Other such proteins include LSm3, a core component of U6 snRNA–protein complexes in spliceosomes,^73^, ^74^ and UPF2 (Supplementary table 1), a key mediator of the nonsense‐mediated mRNA quality control pathways that recruits endonucleases and other factors to regulate aberrant mRNA decay.^75^ Such interactions may enable evolutionarily diverse viruses to manipulate host/virus mRNA or ncRNA biogenesis and stability. The immune mechanisms and potential therapeutic applications of RNA post‐transcriptional control are discussed further by Yoshinaga and Takeuchi^119^ in another article in this Special Feature.

#### Interactions with innate immunity

Toll‐like receptor 7 (TLR7) and TLR8 recognise extracellular ssRNA as short as 3 and 2 nt, respectively, and are activated to a greater extent in a sequence‐specific manner irrespective of end modifications.^76^, ^77^, ^78^ In a similar fashion, TLR3 recognises extracellular as well as endosomal dsRNA longer than 21 nt.^79^, ^80^ The retinoic acid‐inducible gene‐I (RIG‐I)‐like receptors sense intracellular RNA. These include the prototypical RIG‐I, which is activated by ssRNA as short as 10 nt harbouring a di‐ or tri‐phosphorylated 5′ end.^81^ MDA5 is activated by large RNA molecules, while LGP2 recognises RNA as short as 12 nt irrespective of phosphorylation or hydroxylation at the 5′ end. LGP2 activation supports MDA5‐dependent signalling while inhibiting both RIG‐I and Dicer.^82^, ^83^ TLR and RLR activation stimulates IFN‐I expression, which activates JAK‐STAT signalling to modulate expression of hundreds of IFN‐stimulated genes, thereby placing infected cells and local tissues in an antiviral state.^8^ Accordingly, exogenous RNAs including viral RNA, siRNA and their breakdown products are potent stimulators of IFN signalling. Such unwanted immune activation remains a significant challenge in RNA‐based therapeutics. As RNAi processing is further downregulated upon IFN stimulation,^84^ the IFN and RNAi systems compete in a manner detrimental to the efficacy of RNAi‐based therapeutics.

#### Current and future therapeutic applications of RNAi

RNAi‐based approaches to antiviral therapy show both promise and new and familiar challenges. Most known primate‐infective viruses manipulate IFN signalling^85^; however, despite nearly two decades of study, the role of RNAi as a specific immune defence mechanism in somatic, IFN‐responsive tissues remains controversial.^86^, ^87^ If IFN‐ and RNAi‐mediated immunity are incompatible, human‐infective viruses would likely be subjected to only weak, if any, specific RNAi‐mediated immune selective pressure. This suggests the ‘dormant’ immune functions of the mammalian RNAi system could be re‐engineered as a future antiviral or immunotherapeutic strategy. Alternatively, the gut microbiome, which is a crucial regulator of immune homeostasis, T‐cell activation and predicts treatment outcomes in anti‐PD‐1 cancer immunotherapy, might be genetically modified to secrete therapeutic small RNAs.^88^, ^89^, ^90^ RNAi‐based strategies could be used to modulate host immune programme or selectively and reversibly block expression of key multifunctional host proteins in or near virus‐infected tissues, thereby multiply regulating viral replication as discussed earlier.

From the first RNAi patent filing in 1998 until the end of 2017, ~ 8500 siRNA‐based and 2000 miRNA‐based therapeutic patents were filed in the United States. Most were for anticancer applications, followed by viral infections and inflammatory disorders.^91^ At present, the US National Library of Medicine lists 87 ‘miRNA’, 28 ‘siRNA’ and 26 ‘RNAi’ interventional clinical trials as underway or completed. Several trials involved patisiran, which, in 2018, became the first RNAi‐based therapeutic approved by the US FDA. Patisiran is an LNP‐encapsulated siRNA (siRNA‐LNP) delivered to hepatocytes, where it transiently induces RNAi‐mediated silencing of wild‐type and mutant transthyretin mRNA. Prior to infusion, patients receive a combination of oral acetaminophen, intravenous corticosteroid and histamine H1 and H2 receptor antagonists, yet infusion‐related reactions remain one of the most frequent adverse events.^92^ Antagonists to other immune receptors as outlined above may further suppress IFN stimulation by circulating siRNA‐LNPs or their breakdown products. Further reductions in immunogenicity may be achieved through RNA modifications such as pseudouridylation^93^ or by encapsulating siRNAs in exosome vesicles or other biological nanoparticles, which have the added advantage of industrial scale‐up using bioreactors.^94^ An alternative approach may be to again leverage the ability of viral evolution to inhibit host immunity. For example, reversibly incorporating exogenous RNA into ribonucleoprotein complexes, composed of proteins that have evolved to inhibit IFN signalling, could simultaneously yield therapeutic RNA delivery and IFN suppression.

#### Virus‐oriented RNAi therapies

Remarkably, siRNA‐LNP ‘cocktails’ of perfect complementarity to Ebola virus (EboV) RNA have been reported to confer 100% protection in non‐human primates when administered as late as 3 days post‐lethal challenge.^54^ Nevertheless, nucleotide escape mutants and genetic variation between EboV strains in different geographic locations necessitate accurate, up‐to‐date sequencing data on circulating strains in order to continuously generate effective siRNA cocktails. Recently, a protocol employing the MinION portable sequencer was developed that enabled the direct sequencing of an intact RNA virus genome (IAV) for the first time.^95^ Direct sequencing of field EboV strains would drastically reduce the current development time of new siRNA‐LNPs. Nevertheless, further improvements in this sequencing technology will be required for accurate, cost‐effective, routine sequencing of substantially lower‐yielding and genetically diverse field strains.

As with other virus‐oriented treatments, the problem of viral resistance to RNA‐based therapeutics is perhaps best illustrated by HIV. Liu *et al*. produced a double long hairpin RNA (dlhRNA) that was processed endogenously to raise four anti‐HIV shRNAs directed against *gag*,* tat*,* vpu* and *env* transcripts. Despite the cells stably expressing the dlhRNA, together with the combinatorial targeting approach and using virus produced from a single molecular clone, nucleotide escape mutants emerged, integrated and proliferated in as few as 8 days post‐infection.^96^ Notably, however, viral transcript knockdown was variable and incomplete, thereby creating an environment suitable for escape mutant propagation. Selecting RNAi targets in the virus that are even more highly conserved, as well as incorporating a larger number of these in an shRNA ‘cocktail’, may better resist escape mutants and yield longer‐lasting efficacy in future.

#### Host‐oriented RNAi therapies

Virus‐oriented RNA therapies engage the virus on its own terms and in full view of its evolutionary strengths. One alternative approach is to sequester host miRNAs crucial for viral replication. miR‐122 is highly expressed in liver and is necessary for HCV replication. This is targeted by two host‐oriented therapeutics, miravirsen and RG‐101.^97^ Miravirsen showed some efficacy with few adverse effects in clinical trials, while RG‐101 showed promising efficacy but remains subject to clinical hold because of adverse effects.

Besides safety, a clear limitation is that not all medically relevant viruses use host miRNAs as key elements in their replication strategy. One solution is to engineer such viruses to harbour endogenous miRNA‐targeting sequences. This yields recombinant viruses resembling wild‐type but with greatly reduced pathogenicity, restricted tissue tropism and impaired replicative fitness, with potential use as vaccines. Using poliovirus (PV), which replicates primarily in the pharynx and gastrointestinal tract but causes severe neurological disease, Barnes *et al*. first demonstrated that recombinant PV harbouring a complementary sequence of murine brain‐specific miR‐124a was severely compromised in its ability to replicate within this tissue. When this sequence was substituted for a sequence complementary to the ubiquitously expressed miRNA let‐7a, PV replication was further reduced, indicating miRNAs may be used to control tissue tropism. Notably, similar effects were obtained in mice rendered genetically unresponsive to IFN, which nevertheless generated protective antibodies against reinfection by between 10 and 10 000 times the LD_50_ of wild‐type PV.^98^ This approach was recently used by Yee *et al*.^99^ towards developing a live attenuated vaccine for enterovirus 71. Similar results were also obtained by Kelly *et al*.^100^ using Coxsackievirus, where a majority of mice inoculated with recombinant virus harbouring tissue‐specific miRNA‐targeting sequences showing greatly reduced morbidity and mortality up to 70 days post‐infection. Benitez *et al*. showed that mice inoculated with as much as 2500 times the LD_50_ of IAV, also modified to harbour murine miRNA‐targeting sequences, remained asymptomatic up to 10 days post‐infection. These mice were also IFN‐unresponsive, confirming that mammals can, in principle, elicit a highly effective RNAi‐mediated antiviral response and immunological memory against evolutionary diverse viruses in the absence of IFN‐I.^101^

Nevertheless, other viruses harbouring similar modifications have shown mixed results. In contrast to their earlier work on Coxsackievirus, Kelly *et al*. found vesicular stomatitis virus engineered to contain various host miRNA‐targeting sequences largely resisted miRNA‐mediated restriction. Nevertheless, a decrease in neurotoxicity was observed with miR‐125 that also preserved the virus’ oncolytic activity,^102^ properties that are crucial in cancer immunotherapy applications. DenV was successfully restricted from hematopoietic cells by introducing four miR‐142 targeting sites, although the virus quickly reverted and continued proliferating at low levels after excising all four sites.^103^ Since there appears to be no clear pattern that might explain these disparate effects between virus species, additional work remains in order to use recombinant miR‐targeting viruses for routine therapeutic use.

Nearly 20 years elapsed between the first patent filings and the realisation of an approved RNAi‐based therapeutic. While challenges remain, the coming decade appears likely to mark the beginning of the growth curve for creative new approaches to RNA‐based therapeutics for antiviral and immunotherapeutic applications.

### Designer vaccines

To elicit humoral as well as long‐lasting cellular immunity, live attenuated vaccines are the most effective therapy currently available. These are typically produced by passaging viral isolates in permissive immune‐deficient hosts such as embryonated chicken eggs or non‐human continuous cell lines (e.g. Vero), thereby forcing viral re‐adaptation and loss of virulence in the original host. However, the basis for attenuation is usually ill‐defined. Some species or clinical isolates are not readily amenable to current *in vitro* culturing methods (e.g. norovirus),^104^ which often fail to recapitulate essential elements of the viral replication cycle and pathogenesis. Furthermore, attenuated strains are often so compromised that immune adjuvants are required to stimulate antigenicity upon inoculation. Collectively, these challenges increase production costs of many vaccines while limiting detailed studies and the number of viruses for which safe and effective live vaccines can be produced.

To address production costs, RNAi and CRISPR/Cas9 have recently been applied in attempts to engineer cells that produce greater viral yields. Using a genome‐wide RNAi screen in HEp‐2C cells and validation in the Vero cell line approved for vaccine development, van der Sanden *et al*. identified several gene knockdowns that drastically increased yields of multiple PV, enterovirus and rotavirus strains. However, these effects on viral replication were not recapitulated on follow‐up.^105^ As the reasons for these discrepant results remain unclear, challenges evidently remain in engineering cell lines that support greater viral yields for vaccine deployment.

An alternative approach is to genetically re‐program cells derived from the host species that serve as the natural reservoir of the virus. One advantage of this approach is the likely greater amenability of previously uncultivable or poor‐yielding viruses for cultivation *ex vivo*. Additionally, the resulting strain will likely preserve some replicative competence upon inoculation, thereby eliciting stronger immunity in the absence of adjuvants. The challenge remains, however, to identify which parts of the cell or culture methods should be modified to generate broad permissiveness, high titres and greatly reduced virulence simultaneously. Obvious candidates include genes that restrict viral replication but are dispensable for cell survival, such as IFN genes and their signalling components, and potentially certain multifunctional proteins. Taken to its logical conclusion, it should be possible to genetically engineer an immune‐null human cell substrate within which to passage virus free of virulence factor targets and immune selective pressure. In this way, the strain that emerges will likely exhibit strong antigenicity but severe degradation in mechanisms of host immune antagonism. Such an approach may also prove useful in the context of viruses that cause severe disease primarily through cytokine hyperactivation.

Additional modifications to this host‐oriented approach may further improve vaccine yield or safety. These may include eliminating pro‐apoptotic genes to limit virus‐induced programmed cell death and increase viral titres. The culture system itself may be improved to better represent the three‐dimensional microarchitecture of the host tissue and other features necessary for efficient viral replication. Organoids and other stem cell‐derived tissues represent one approach under recent and intensifying examination. Human lung organoids have been demonstrated to recapitulate key properties of RSV pathogenesis,^106^ and human intestinal epithelium has been developed for previously uncultivable norovirus.^104^ Additionally, incorporating multiple tissue‐specific miRNA‐targeting sequences within the attenuated viral genome may improve vaccine safety by impairing its ability to replicate within inflammation‐sensitive or irreplaceable tissues. Inversely, this same strategy may be used to help guide infection of certain tissues such as oncolytic viruses in the case of cancer immunotherapy.^100^ Another safety feature could involve a drug‐selective ‘kill switch’, whereby key viral proteins are fused to the FK506‐binding protein 12 destabilisation domain. Viral fusion proteins are ‘rescued’ from proteasomal degradation in the presence of the drug Shield‐1 but efficiently degraded upon its removal,^107^ thereby yielding a conditionally replication‐incompetent strain. The coming decade appears likely to see two key transitions: from empirical to designer vaccines, and from viruses as pathogens to important tools in biotechnology.

### Neo‐virology and future biotechnologies

Of the 8.7 million known species on earth, viruses are likely the most ancient and prolific with at least 10^31^ virions estimated to exist today.^10^ Sampling only a fraction of these diverse host–virus interactions has already resulted in ground‐breaking biotechnologies including biomolecule and bioactive compound delivery systems, RNAi‐mediated antiviral therapies and genetic engineering using zinc finger nucleases, TALENs and CRISPR/Cas9. These have wide‐ranging applications in antiviral therapy and vaccine development, immunotherapy, regenerative medicine, environmental science and numerous other fields.

Neo‐virology is an emerging field aiming to further this trajectory of innovation by systematically characterising the roles of viruses and viral‐mediated processes in the entire living biosphere.^10^ As the unexplored genetic diversity of viruses is unlocked through improvements in sequencing technologies and big data analysis, the molecular basis of host–virus interactions and the evolutionary relationships between highly divergent species are becoming clearer.

One area of interest is the increasing number of nucleocytoplasmic large DNA viruses (NCLDVs) being discovered in prokaryote, protist and invertebrate hosts and in soil and water. These include two amoebal pathogens: pandoravirus, which harbours the largest known viral genome at 2.5 Mb,^108^ and mimivirus, an emerging human pathogen harbouring a 1.2 Mb genome.^109^ In stark contrast to RNA viruses, these giant DNA viruses appear capable of acquiring additional information without clear bound, presenting an alternative solution to the information economy paradox. These viruses are also suggested to readily generate genes *de novo*.^13^ While few complete genome sequences of such viruses are currently available, most of the numerous proteins encoded by these vast viral genomes are entirely novel.^108^, ^110^, ^111^ If the process for *de novo* generation of viral genes can be harnessed, this could support efforts at directed evolution of new and useful biological functions.

Nucleocytoplasmic large DNA viruses proteins with inferred functions reveal interesting patterns. A recently discovered NCLDV encodes a full set of eukaryote‐like histones and a DNA polymerase, potentially placing it at the root of the eukaryotic clades.^110^ An ancient NCLDV‐like virus may have been responsible for the origin of the eukaryote nucleus itself.^112^ Subsequently, eukaryote multicellularity, coupled with programmed cell death, may have emerged as an ancient antiviral defence mechanism,^113^ possibly enabling the rise of complex life. Retroviral elements, in addition to driving formation of the mammalian placenta, control hormones involved in gestation and birth timing in some mammals.^114^ Such elements comprise ~ 8% of the human genome, which also contains genetic material derived from viruses with no retro‐transcription or integration functionality.^115^ Thus, despite being strictly non‐living, viruses have radically shaped the living biosphere. Understanding these processes could enable new approaches to control the basic functions of life.

Areas where improved understanding of such host–virus interactions could have immediate implications include human disease, bioremediation of harmful algal blooms and climate change. Contemporary viruses overwhelmingly infect marine microorganisms, turning over an estimated 20% of the ocean microbiome daily.^9^ These infections have significant effects on carbon absorption by oceans as well as global nutrient and energy cycles.^11^, ^14^ Nevertheless, the interactions between the ocean virome and microbiome and their effects climate remain poorly characterised. By examining evolutionarily diverse host–virus interactions in detail, immunology and virology may provide effective solutions to not only human disease but also cost, environmental and other sustainability issues of our time.

## Concluding remarks

As outlined in this review, current antivirals almost exclusively target virus proteins and have significant development costs, limited therapeutic range and are ultimately susceptible to escape mutant selection. Despite being intractably limited in informational size, RNA viruses are thorough problem solvers, often subverting multitasking host proteins to achieve favorable host subversion at minimal informational cost. Such solutions to the viral information economy paradox are often conserved, creating opportunities to leverage imbricated multidependency on key host proteins for host‐oriented antiviral therapies that are more effective, broad‐acting and ultimately more cost‐effective. Although such proteins can present challenging therapeutic targets, host‐oriented therapies will synergise with increased therapeutic drug availability and developments in RNAi, precision medicine and immunotherapy. Additionally, methods of increasing viral antigenicity yet controlling replication and tissue tropism will increase the number of viruses for which safe and highly effective vaccines can be produced. Viruses such as NCLDVs appear to readily acquire new information with which to subvert their hosts. The fruits of problem‐solving by such viruses include large numbers of proteins with unique and unknown biological functions. The influence and genetic hallmarks of viruses at both extremes are found in humans and throughout the living biosphere. By expanding the examination of evolutionarily diverse host–virus interactions, disease, cost, environmental and other sustainability issues of our time may be remedied by leveraging, rather than yielding to, the properties of RNA and DNA viruses as developed through co‐evolution with their hosts.

## Conflict of interest

The author declares no conflict of interest.
